# Why Nurses Intend to Override AI Alerts: How Alert Fatigue, Moral Distress, and Team Psychological Safety Shape Self-Reported Trust Calibration Toward Clinical Decision Support

**DOI:** 10.3390/healthcare14142063

**Published:** 2026-07-09

**Authors:** Emilia Clej, Camelia Fizedean, Adelina Gherman, Adrian Cosmin Ilie, Melania Lavinia Bratu, Felicia Marc

**Affiliations:** 1 Doctoral School, “Victor Babes” University of Medicine and Pharmacy, Eftimie Murgu Square 2, 300041 Timisoara, Romania; emilia.clej@umft.ro; 2 Centre for Translational Research and Systems Medicine (CERT-MEDS), “Victor Babes” University of Medicine and Pharmacy, 300041 Timisoara, Romania; 3 Faculty of Nursing, “Victor Babes” University of Medicine and Pharmacy, 300041 Timisoara, Romania; fizedean.camelia@umft.ro; 4 Multidisciplinary Heart Research Center, “Victor Babes” University of Medicine and Pharmacy, 300041 Timisoara, Romania; 5 Department of Functional Sciences, Discipline of Public Health, Center for Translational Research and Systems Medicine, “Victor Babes” University of Medicine and Pharmacy, 300041 Timisoara, Romania; ilie.adrian@umft.ro; 6 Center for Neuropsychology and Behavioral Medicine, Discipline of Psychology, Faculty of General Medicine, “Victor Babes” University of Medicine and Pharmacy, 300041 Timisoara, Romania; 7 Department of Medical Sciences, Faculty of Medicine and Pharmacy, University of Oradea, 410073 Oradea, Romania; fmarc@uoradea.ro

**Keywords:** decision support systems, clinical, burnout, professional, moral distress, nursing staff, hospital, artificial intelligence

## Abstract

**Background and Objectives:** Hospitals increasingly use AI tools that give nurses on-screen alerts and recommendations (AI-supported clinical decision support, AI-DSS). When nurses override these alerts too often, useful guidance can be lost; when they trust them blindly, errors can slip through. We examined which work and wellbeing factors are associated with nurses’ self-reported intention to override AI alerts, rather than observed override behavior. **Methods:** We surveyed 239 registered nurses (76.6% female; mean age 33.7 years) at a large hospital in Timișoara, Romania, from January to March 2025. Questionnaires measured alert fatigue, moral distress, mental workload, sleep problems, resilience, team psychological safety, and how strongly nurses intended to override AI alerts. **Results:** Nurses fell into three groups: those who tended to over-trust AI (26.8%), those with balanced trust (41.0%), and those who resisted it (32.2%). The resistant group had the strongest intention to override alerts and the weakest sense of psychological safety. Alert fatigue was the factor most strongly associated with override intention, and this association was partly accounted for by moral distress. The indirect association was weaker among nurses reporting higher team psychological safety. An exploratory model using these factors distinguished nurses with high self-reported override intention with acceptable accuracy. Because all variables were measured at a single time point, findings are associative and hypothesis-generating rather than causal. **Conclusions:** How nurses respond to AI alerts depends less on the technology than on their workload, ethical strain, and team climate. Cutting unnecessary alerts, easing moral distress, and building psychological safety may help nurses use AI more safely.

## 1. Introduction

The integration of artificial-intelligence-supported clinical decision support (AI-DSS) into hospital nursing workflows has shifted attention from technical performance toward the human factors that determine whether algorithmic outputs are trusted, ignored, or selectively overridden at the bedside. Across the recent literature, the phenomenon of algorithm aversion—the systematic preference for human over machine judgment even when the latter performs comparably or better—has emerged as a critical implementation barrier [1], while its counterpart, algorithm appreciation, describes uncritical reliance on automated outputs [2]. Both extremes are clinically problematic. Nurses sit at the interface between continuous patient observation and digital prompts, and their override decisions can either correct algorithmic blind spots or, conversely, introduce inconsistent care. Understanding the antecedents of override behavior is therefore central to the safe deployment of AI-DSS in nursing practice.

Alert fatigue, defined as the desensitization that follows repeated exposure to clinical prompts of variable specificity, has been associated with delayed responses, missed prioritization, and reflexive dismissal of decision-support outputs [3]. In digitally intensive units, the cumulative burden of pop-ups, scoring nudges, and documentation reminders may exceed the cognitive capacity required for meaningful interpretation, especially when alerts compete with bedside care, and prior work has shown that workload, work complexity, and repeated alerts together amplify the desensitization effect [4]. The transition from rule-based alerts to AI-generated risk scores has not resolved this burden but rather reshaped it, since nurses are now asked to weigh probabilistic outputs they did not generate against their own situational awareness [5]. Whether alert fatigue specifically drives override intention toward AI-DSS recommendations, independent of generalized burnout, remains insufficiently characterized.

Moral distress—the psychological burden experienced when nurses recognize the ethically correct action but feel constrained from performing it [6]—has long been linked to attrition and reduced quality of care. The growing presence of AI in clinical workflows adds new sources of moral distress, including disagreement with algorithmic prioritization, perceived erosion of professional judgment, and concerns about accountability when recommendations are followed or overridden [7]. These ethical tensions may be especially relevant for nurses, whose scope of practice emphasizes relational care and continuous observation. Moral distress may therefore mediate part of the relationship between digital workload and downstream override behavior, particularly when nurses feel that compliance with algorithmic suggestions would conflict with their professional standards.

Psychological safety—the shared belief that team members can voice concerns, question decisions, and admit uncertainty without fear of negative consequences [8]—has emerged as a foundational determinant of safe clinical performance. In the context of AI-DSS, psychological safety likely shapes whether nurses feel authorized to challenge algorithmic recommendations on the basis of their bedside assessment, and a recent synthesis of healthcare-team literature has identified leadership behaviors and structured speak-up routines as central enablers of this climate [9]. In low-safety climates, override behavior may become reflexive or covert, whereas high-safety environments may allow more deliberate, calibrated decisions. Few studies, however, have examined psychological safety as a moderator of the alert fatigue–override pathway, despite a strong conceptual rationale for an interaction effect on trust calibration.

Trust calibration toward algorithmic systems is the alignment between perceived and actual reliability of the system, and it is a key construct in human-factors research on automation [10]. Three calibration patterns are typically observed: over-trust (algorithm appreciation), under-trust (algorithm aversion), and calibrated trust, each producing distinct patterns of automation use, misuse, disuse, and abuse [11]. Each pattern carries distinct clinical implications. Algorithm appreciators may comply with recommendations that should be questioned; algorithm-averse nurses may dismiss outputs that would have improved care; calibrated nurses use AI-DSS as one input among several. Identifying these phenotypes within a single nursing cohort, and characterizing their work-system and psychological correlates, may inform targeted interventions rather than uniform deployment strategies.

Although the recent nursing literature has explored individual constructs—alert fatigue, burnout, digital readiness, AI acceptance—few investigations have simultaneously examined alert fatigue, moral distress, psychological safety, and trust calibration as interlocking determinants of AI-DSS override intention. Broader analyses of the convergence between human and artificial intelligence in medicine emphasize that successful integration depends as much on the surrounding work system and human-factors architecture as on algorithmic performance [12]. Evidence from Central and Eastern European hospitals, where digital transformation continues at varying rates, is particularly limited. Accordingly, the present study had four aims: (1) to characterize AI-DSS override intention and identify latent work-system profiles indicative of trust-calibration tendencies among hospital nurses; (2) to estimate the independent associations of alert fatigue, moral distress, cognitive load, psychological safety, resilience, and sleep disturbance with override intention; (3) to test whether psychological safety moderates the alert fatigue–override association through moral distress; and (4) to evaluate, in an exploratory fashion, the discrimination and clinical utility of a multivariable model for distinguishing nurses with high self-reported override intention.

Four directional hypotheses were specified. First, higher alert fatigue is associated with higher self-reported override intention (H1). Second, higher moral distress is associated with higher override intention and accounts for part of the alert fatigue–override intention association (H2). Third, higher team psychological safety is associated with lower override intention and attenuates the indirect association operating through moral distress, such that the indirect association is weaker at higher levels of psychological safety (H3). Fourth, as an exploratory aim, a multivariable model combining work-system and psychological indicators identifies nurses with high versus lower self-reported override intention (H4). The hypothesized ordering is grounded in job demands–resources and conservation-of-resources frameworks, in which chronic exposure to demanding stimuli such as repeated, variably specific alerts depletes regulatory resources and generates ethical strain, which in turn shapes defensive dispositions toward the demanding stimulus. Psychological safety is positioned as a moderator rather than a mediator because it is a comparatively stable team-climate property expected to condition how individual strain is expressed rather than to be produced by alert fatigue.

## 2. Materials and Methods

### 2.1. Study Design and Setting

This cross-sectional, single-center study was conducted at a 1140-bed tertiary care hospital in Timișoara, Romania, between January and March 2025. The institution functions as a referral center for the western region of the country and operates a hybrid digital environment in which an integrated electronic health record is used alongside emerging AI-supported tools for risk stratification, prioritization, and documentation assistance. At the time of data collection, AI-DSS deployment was incremental rather than fully embedded, providing an analytically valuable transition window in which nurses had been exposed to algorithmic prompts in some workflows while retaining substantial reliance on conventional clinical judgment. Eligible participants were registered nurses with at least six months of continuous practice in inpatient units (critical care or general wards). Nurses on extended leave (>30 days), agency staff, and nurses working exclusively in outpatient settings were excluded.

Three categories of AI-supported functionality were active within the electronic health record during the study period: a deterioration risk-stratification module generating color-coded patient risk scores and threshold-based escalation prompts; a prioritization aid that re-ordered task and observation lists according to algorithmically estimated acuity; and documentation-support features providing auto-generated text suggestions and completeness reminders. These tools surfaced to nurses as on-screen scores, banner prompts, and pop-up reminders, and did not replace standing clinical protocols. The modules were enabled progressively and unevenly across units. Forty-seven nurses (19.7%) reported prior structured exposure to AI-DSS tools, defined as having received formal orientation or training on at least one module; the remainder encountered the tools during routine practice without formal orientation. System-level logs quantifying per-nurse alert frequency, alert-type distribution, or override events were not available for linkage to survey responses.

Reporting followed the STROBE guideline for observational studies [13]. The unit of analysis was the individual nurse. Recruitment was coordinated through unit nursing leadership, who disseminated the questionnaire link and printed forms during scheduled handover meetings; participation was voluntary and uncompensated. Of 271 nurses approached, 239 returned complete questionnaires (response rate 88.2%). To minimize self-selection effects, no individual-level reminders were issued; instead, two ward-level reminder rounds were conducted. The sampling frame deliberately captured both day-only and rotating/night-shift personnel because shift configuration was hypothesized to differentially affect alert fatigue and sleep disturbance.

### 2.2. Ethical Considerations and Data Collection Procedure

The study protocol was approved by the Local Commission of Ethics of the “Pius Brinzeu” Clinical Emergency Hospital in Timișoara, Romania, and all procedures were conducted in accordance with the Declaration of Helsinki and applicable Good Clinical Practice principles. Before participation, nurses received a structured information sheet describing the study aims, the voluntary nature of participation, the absence of supervisor access to identifiable responses, and their right to withdraw at any point without consequence. Written informed consent was obtained from all participants. To minimize social desirability bias and response inhibition, questionnaires were completed privately in unsupervised areas, responses were collected anonymously, and unit managers had access only to aggregated unit-level summaries.

Data were collected via a structured, self-administered questionnaire offered in two equivalent formats: a secure web-based version and a paper version, with each respondent allowed to choose. The questionnaire was pilot-tested for clarity and completion time on a separate convenience sample of 12 nurses outside the target cohort; mean completion time was 21.4 min. To reduce common-method bias at the design stage, conceptually distinct constructs were measured using separate validated instruments, items were presented in randomized blocks where feasible, and three procedural attention-check items were embedded. Five respondents who failed two or more attention checks were retained but flagged for sensitivity analysis; their exclusion did not materially affect estimates.

### 2.3. Measures and Instruments

The instrument battery captured digital workload, psychological resources, ethical strain, and AI-related attitudes. Alert fatigue was assessed using a 12-item adapted alert-fatigue scale covering perceived alert volume, perceived alert relevance, response delay, and reflexive dismissal; items were scored on a five-point Likert scale, yielding a total score between 12 and 60 with higher values indicating greater alert fatigue. Moral distress was measured using a 10-item adaptation of the Moral Distress Scale–Revised (MDS–R) focused on conflicts between professional judgment and external constraints, including those introduced by digital workflows. Cognitive load was captured using a six-item brief NASA-TLX-derived instrument tailored to clinical shift demand. Sleep disturbance over the past month was assessed using a brief five-item sleep quality instrument anchored on the Pittsburgh Sleep Quality Index dimensions.

Psychological safety at the unit level was measured using the seven-item Edmondson Psychological Safety Scale, capturing perceived freedom to voice concerns, admit errors, and challenge prevailing decisions, including algorithmic prompts. Resilience was measured using the 10-item Connor–Davidson Resilience Scale (CD-RISC-10) [14]. AI-DSS override intention—the primary outcome—was operationalized through an eight-item scale assessing nurses’ stated intention to override, dismiss, or modify AI-generated recommendations across structured scenarios, with higher scores indicating stronger override intention. Trust-calibration tendency was assessed with a complementary five-item instrument capturing the perceived appropriateness of accepting versus questioning algorithmic outputs across clinical scenarios. Internal consistency of all multi-item scales was evaluated using both Cronbach’s α and McDonald’s ω, and, for the adapted and newly developed instruments, composite reliability (CR) was additionally computed from the standardized confirmatory factor loadings. McDonald’s ω was estimated because it does not assume tau-equivalence and provides a more accurate lower-bound estimate of reliability than α for scales with heterogeneous item loadings. Across all instruments, ω was closely aligned with α and ranged from 0.76 to 0.88, and CR for the four adapted or newly developed scales ranged from 0.79 to 0.87, indicating acceptable-to-good reliability by conventional thresholds (ω and CR ≥ 0.70). The survey also captured age, sex, education, years of experience, unit type, shift configuration, and prior structured exposure to AI-supported tools.

The Edmondson Psychological Safety Scale and the CD-RISC-10 were administered in their validated forms without item modification. The remaining four instruments—the alert-fatigue scale, the moral-distress adaptation, the AI-DSS override intention scale, and the trust-calibration tendency scale—were adapted or developed for this study. All instruments were translated into Romanian using a forward–backward procedure: two independent bilingual nurse-researchers produced forward translations, a third bilingual translator blind to the source produced the back translation, and discrepancies were reconciled by consensus with a clinical psychologist. Content validity was evaluated by an expert panel of six clinicians and methodologists who rated each item for relevance on a four-point scale; the item-level content validity index (I-CVI) ranged from 0.83 to 1.00 and the scale-level content validity index (S-CVI/Ave) ranged from 0.89 to 0.96. Comprehensibility and completion time were confirmed in the 12-nurse pilot.

The adapted alert-fatigue scale was not taken from a single pre-existing validated instrument but was assembled as a composite adaptation drawing on the operational definitions and item content of three prior sources on clinical reminder and alert fatigue [3,4,5]. The constructs and candidate wording were informed by the scoping and systematic reviews of Hamblin et al. [3] and Backman et al. [5] and by the alert-fatigue operationalization of Ancker et al. [4], from which four target facets—perceived alert volume, perceived alert relevance, response delay, and reflexive dismissal—were defined. Because no single validated alert-fatigue scale spanning these four facets was available for the AI-DSS context, items were written to represent each facet, harmonized in wording, and then subjected to the expert-panel content-validity and pilot procedures described below. From an initial pool of 16 candidate items, four were removed after pilot review for redundancy or low clarity, yielding 12 items across the four facets; no items were added beyond these conceptual domains. The resulting instrument should therefore be regarded as a study-specific adaptation assembled from multiple validated sources rather than a direct application of a single previously validated scale. A representative item is “I find myself dismissing on-screen alerts without fully reading them”. The moral-distress measure was a 10-item adaptation of the Moral Distress Scale–Revised (MDS–R) [6] in which constraint sources were specified to include digital and algorithmic workflows (e.g., “I feel pressured to follow a system-generated recommendation that conflicts with my clinical judgment”); the response format was retained and no new constructs were introduced. The eight-item AI-DSS override intention scale was newly developed: each item presents a standardized clinical scenario in which an AI-DSS prompt appears, and respondents rate on a five-point scale their likelihood of overriding, dismissing, or modifying the recommendation (e.g., “A risk score flags a stable patient as high-risk; I would dismiss the alert and proceed with my plan”). The five-item trust-calibration tendency scale was likewise newly developed, capturing the perceived appropriateness of accepting versus questioning algorithmic outputs. Items for both new scales were generated from the human-automation trust literature [10,11], refined through the expert panel, and pilot-tested. 

Dimensionality of the two newly developed scales was examined by splitting the sample at random and conducting exploratory factor analysis in one half and confirmatory factor analysis in the other. The AI-DSS override intention scale supported a single-factor solution (first-factor eigenvalue 3.9, explaining 49% of variance; confirmatory fit CFI = 0.96, TLI = 0.94, RMSEA = 0.061, SRMR = 0.045; standardized loadings 0.52–0.78). The trust-calibration tendency scale also supported a single-factor solution (CFI = 0.97, RMSEA = 0.058, SRMR = 0.041; loadings 0.49–0.74). The intention-to-leave indicator was measured with a single item (“How likely are you to leave your current nursing position within the next 12 months?”) rated on a five-point scale and dichotomized at the upper two response categories (“likely” or “very likely”) for descriptive reporting; it was a pre-specified secondary descriptive variable.

### 2.4. Statistical Analysis

Analyses were performed in R version 4.3.2 (R Foundation for Statistical Computing, Vienna, Austria) with the lavaan, tidyLPA, mediation, and rmda packages. Continuous variables approximately normally distributed were summarized as mean ± SD and compared using Student’s *t*-tests or one-way ANOVA with trend tests across ordered categories; non-normal continuous variables were summarized as median (IQR) and compared using Mann–Whitney U or Kruskal–Wallis tests. Categorical variables were summarized as counts and percentages and compared using χ^2^ tests or Fisher’s exact tests when expected cell counts were below five. Pearson correlations quantified pairwise associations among continuous study variables. Multivariable linear regression modeled AI-DSS override intention as a continuous outcome adjusted for age, sex, unit type, shift configuration, education, and years of experience; multicollinearity was screened via variance inflation factors with a threshold of 4.0. A multinomial logistic regression evaluated correlates of trust-calibration phenotype membership, with the calibrated phenotype as reference.

Latent profile analysis (LPA) was used to identify latent work-system profiles from standardized scores of alert fatigue, moral distress, cognitive load, psychological safety, resilience, and sleep disturbance. Trust-calibration tendency and override intention were not entered as profile indicators; profiles were defined solely by the six work-system and psychological variables and were subsequently cross-tabulated with the independently measured trust-calibration tendency and override intention scores to characterize each class. Models with 2–5 profiles were compared using the Bayesian information criterion (BIC), sample-size adjusted BIC (SABIC), entropy, and the bootstrapped likelihood ratio test (BLRT); the three-profile solution was selected on combined statistical fit and clinical interpretability. For each retained profile, the number of cases, proportion, and mean posterior (assignment) probability were reported. Correlates of profile membership were examined with multinomial logistic regression; because the predictors in that model are the same variables used to estimate the profiles, the model was specified as a descriptive characterization of class separation rather than a test of independent correlates, and was additionally estimated with the three-step (BCH) approach that accounts for classification uncertainty. Moderated mediation analyses with 5000 bootstrap resamples tested whether moral distress and psychological safety statistically accounted for the alert fatigue–override intention association, and whether psychological safety moderated this indirect path, following the conditional process analytic framework of Hayes [15]. An exploratory Gaussian graphical model with the graphical LASSO procedure was used to estimate the partial correlation network among the eight principal variables, with node centrality summarized by strength, closeness, and expected influence and bootstrap edge stability quantified using the case-dropping CS-coefficient, supplemented by non-parametric bootstrap confidence intervals for edge weights and by edge-difference and centrality-difference tests. Discrimination of a multivariable model for high override intention (top tertile) was evaluated with a c-statistic and exploratory decision-curve analysis using 10-fold internal cross-validation. The multivariable linear regression and the moderated mediation model corresponding to hypotheses H1–H3 were pre-specified as confirmatory analyses; the latent profile analysis, the partial correlation network, and the prediction and decision-curve model (H4) were pre-specified as exploratory. A sensitivity analysis restricted to nurses with prior structured AI-DSS exposure was performed for the principal models. All tests were two-sided, with *p* < 0.05 considered statistically significant; given the partly exploratory nature of profile and network analyses, results were interpreted cautiously and as hypothesis-generating rather than confirmatory.

**Figure 1:**
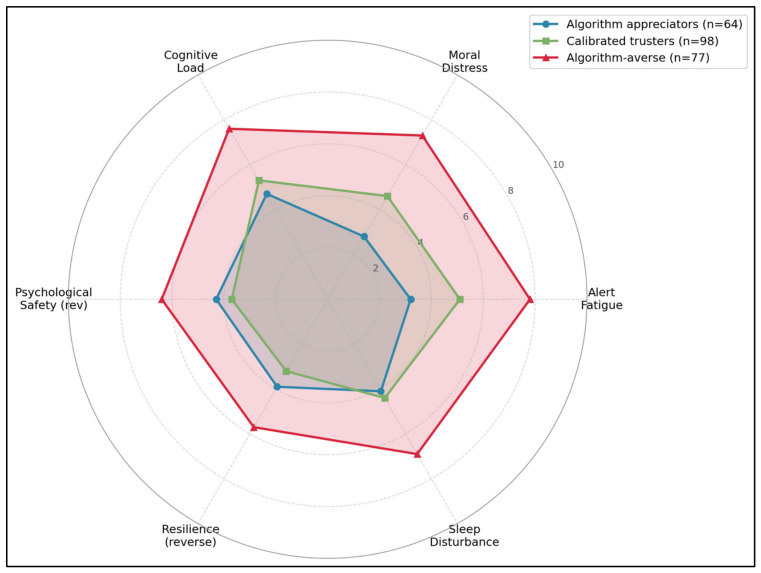
Standardized work-system and psychological profiles across AI-DSS trust-calibration phenotypes.

**Figure 2:**
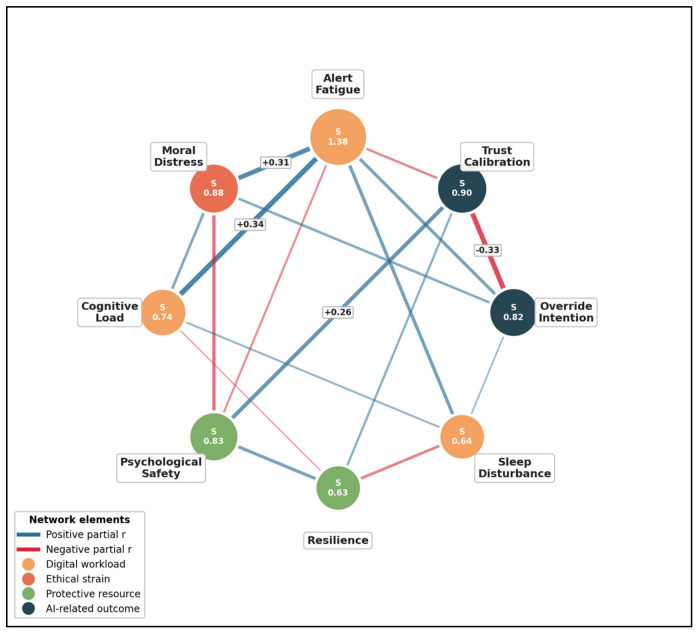
Partial correlation network among the eight principal study variables (*n* = 239), with node strength centrality and bootstrap stability indicators.

In Figure 1, each line represents one latent profile, and the vertical axis shows the group mean of each indicator expressed as a sample-standardized z-score, so that values above zero denote above-average burden or below-average resources relative to the cohort. In Figure 2, nodes represent variables and edges represent the partial correlation between two variables conditional on all others; edge thickness encodes association strength, blue and red denote positive and negative associations, and the graphical LASSO penalty shrinks weak edges toward zero. Node strength summarizes the magnitude of each variable’s connections within the network.

## 3. Results

Of the 239 nurses, 147 (61.5%) worked day-only shifts and 92 (38.5%) worked rotating or night shifts (Table 1). The two groups were similar in sex and education, but rotating/night-shift nurses were slightly younger and less experienced. They also reported clearly worse scores on every key measure: more alert fatigue, more moral distress, lower psychological safety, more sleep problems, and about 24% higher intention to override AI alerts (all *p* < 0.05). Prior structured AI-DSS exposure did not differ significantly between shift groups (*p* = 0.083). These unadjusted comparisons describe differences by shift configuration only; adjusted associations are reported below.

Table 2 reports the reliability and distribution of the eight questionnaires. All scales were reliable, with Cronbach’s α from 0.74 to 0.87 and McDonald’s ω from 0.76 to 0.88; composite reliability for the four adapted or newly developed instruments ranged from 0.79 to 0.87. The two reliability coefficients were consistent for every scale, and no instrument fell below the 0.70 threshold. Most scores sat near the middle of their possible range and were close to normally distributed, supporting the planned analyses. Override intention was the most right-skewed (0.62), meaning many nurses scored low while a smaller group scored high—an early sign of distinct nurse subgroups.

Splitting nurses into low, medium, and high alert-fatigue groups revealed a clear step-by-step pattern (Table 3). As alert fatigue rose, so did moral distress, cognitive load, sleep problems, and override intention, while psychological safety, resilience, and balanced trust all fell (*p*-trend < 0.001 for most). Override intention doubled from the low to the high group (9.4 to 18.9), and the share of nurses reporting their intention to leave within 12 months (Section 2.3) climbed from 13.4% to 42.3%.

Table 4 shows how the eight main variables correlate. Alert fatigue was positively linked to moral distress, cognitive load, sleep problems, and override intention, and negatively linked to psychological safety, resilience, and balanced trust. The strongest single relationship was the negative link between balanced trust and override intention (r = −0.41), indicating that these two attitudes pull in opposite directions. Psychological safety tracked positively with resilience and balanced trust, suggesting team climate shapes both wellbeing and AI attitudes.

The regression model explained 34% of the variation in override intention (Table 5). After adjustment, the strongest independent predictors of greater override intention were alert fatigue, moral distress, low psychological safety, and cognitive load. Resilience and sleep problems mattered less but were still significant. Working in a critical care unit was also associated with higher override intention. Once these work and wellbeing factors were accounted for, demographics (sex, age, education) and shift type were no longer significant predictors, implying that shift effects act mainly through fatigue, distress, and sleep. All multicollinearity checks were acceptable.

Table 6 shows what predicted belonging to each nurse group, using the balanced-trust group as the reference. The model classified nurses well above chance (62% vs. 33%). Nurses with higher alert fatigue, moral distress, cognitive load, and sleep problems were more likely to be in the AI-resistant group, while higher psychological safety was protective. For the over-trusting group, only lower alert fatigue stood out as a distinguishing factor. Because these predictors are the same variables used to derive the profiles, the estimates describe class separation rather than independent correlates of membership; a three-step (BCH) analysis accounting for classification uncertainty produced a similar pattern.

Figure 1 shows the standardized profiles of the three nurse groups across six work and wellbeing dimensions, where higher values mean greater burden or fewer resources. The AI-resistant group scored highest on every dimension, the over-trusting group scored lowest, and the balanced-trust group fell in between. These differences were statistically significant overall (Pillai’s trace = 0.41; *p* < 0.001), and the resistant group differed significantly from both other groups on every dimension. Profile labels were assigned after estimation by cross-tabulation with the independently measured override intention and trust-calibration tendency scores: the high-burden profile showed the highest override intention and lowest trust-calibration tendency (algorithm-averse), the low-burden profile showed the opposite pattern (over-trusting), and the intermediate profile fell between them (balanced-trust). As the profiles were derived from work-system and psychological indicators rather than a direct calibration metric, they are described as work-system profiles aligned with these trust-calibration tendencies.

Table 7 compares latent profile models with 2–5 groups. The three-group solution gave the best balance of statistical fit (lowest BIC and SABIC, highest entropy at 0.78) and clinical sense. Adding a fourth or fifth group did not improve fit and produced very small, unstable groups. Classification was confident (mean within-group probability 0.89). The three profiles comprised the over-trusting class (*n* = 64; 26.8%; mean posterior probability 0.91), the balanced-trust class (*n* = 98; 41.0%; mean posterior probability 0.88), and the algorithm-averse class (*n* = 77; 32.2%; mean posterior probability 0.90); diagonal classification probabilities all exceeded 0.85. The three groups were therefore labeled as over-trusting, balanced-trust, and AI-resistant nurses, shown in Figure 1.

Table 8 reports the decomposition of the cross-sectional association between alert fatigue and override intention. Both component associations were statistically significant, and moral distress accounted for about one-third (33.6%) of the total association, with the remainder represented as a direct association. The indirect association varied with psychological safety, being larger at low psychological safety (0.142) and smaller at high psychological safety (0.041); the index of moderated mediation was statistically significant. As all variables were measured concurrently, these estimates describe associations and do not establish directional effects.

Table 9 reports an exploratory analysis of how well these factors identify nurses with high self-reported override intention, defined as the upper tertile of the override intention scale. Using 10-fold internal cross-validation, the model showed acceptable discrimination (c-statistic 0.78) and good calibration, and a decision-curve analysis indicated positive net benefit relative to treat-all or treat-none strategies across thresholds of approximately 0.08 to 0.36. These estimates derive from internal validation in a single center and are exploratory only.

Figure 2 maps how the eight variables relate to one another once all other variables are held constant. Blue lines show positive links and red lines negative ones, with thicker lines meaning stronger links. The clearest negative link was between balanced trust and override intention, and the strongest positive links connected alert fatigue with cognitive load and moral distress. Alert fatigue was the most connected variable in the network, indicating its central role. Edge weights were estimated with acceptable precision on non-parametric bootstrap, and node-strength centrality was stable (case-dropping CS-coefficient = 0.59). Edge-difference and centrality-difference tests indicated that several individual edges and centrality contrasts were not statistically distinguishable.

## 4. Discussion

### 4.1. Analysis of Findings

The present findings converge on three principal observations, which are associative given the cross-sectional design. First, AI-DSS override intention among hospital nurses was not uniformly distributed but rather concentrated in a definable work-system profile—the algorithm-averse cluster—that combined high alert fatigue, elevated moral distress, low psychological safety, and disturbed sleep. Second, the cross-sectional association between alert fatigue and override intention was partly accounted for by moral distress, and the strength of this indirect association varied with psychological safety, consistent with team climate being related to how digital workload and algorithmic skepticism co-occur. Third, the relevant correlates of override intention are largely modifiable: alert-fatigue burden, moral-distress triggers, and a psychological-safety climate are all amenable to organizational intervention, in contrast to fixed demographic factors that were not independently associated after adjustment. These observations are consistent with prior nursing work in which digital strain and eHealth competence have been shown to track together within the same cohort [16], and they extend recent reports describing the close coupling of technostress with burnout in hospital nurses [17]. The phenotype-based perspective additionally aligns with emerging behavioral models in which nurses’ intention to adopt or resist AI tools depends on the interplay between perceived job stress, social influence, and human–machine trust rather than on technological exposure alone [18]. These findings reframe override behavior less as a property of the nurse and more as a property of the work system in which the nurse functions.

A conceptual distinction is central to the present work. Override behavior denotes documented acts of dismissing, modifying, or accepting specific algorithmic recommendations, whereas override intention denotes a behavioral disposition—the strength of a nurse’s stated likelihood of overriding alerts across standardized scenarios. Behavioral intention is a recognized antecedent of behavior but is not equivalent to it, since intention may diverge from action under situational, workflow, and patient-care constraints. The construct examined here is self-reported override intention.

Our results are broadly consistent with emerging literature on algorithm aversion in healthcare. Recent systematic reviews of nursing students and practicing nurses have documented heterogeneity in clinician acceptance of AI-supported recommendations and have emphasized digital literacy, readiness, and adoption intentions as critical implementation parameters [19]. Reviews of eHealth literacy among hospital providers reach similar conclusions, identifying digital competence as a foundational determinant of safe technology use [20]. The mediating role of moral distress also reinforces theoretical models proposing that ethical conflict, when sustained, shifts nurses toward defensive cognitive strategies, including reflexive override behavior; this interpretation is supported by recent narrative and integrative reviews of AI applications in nursing that highlight how unresolved ethical tensions can compromise both adoption and care quality [21]. The observed moderation by psychological safety is conceptually aligned with broader human-factors frameworks emphasizing the joint design of work, technology, and team climate [22], and with persistent evidence that electronic-health-record-related unintended consequences arise predominantly at the interface between technology and the surrounding work system [23]. Collectively, these strands of literature support an organizational rather than purely educational intervention model and reinforce the interpretation that algorithm aversion in nursing co-occurs with a coherent profile of digital, ethical, and team-climate strain rather than reflecting an isolated cognitive bias.

From an implementation perspective, three tentative directions are suggested by these associations. Reducing alert-fatigue burden through alert pruning, contextualization, and tiered escalation pathways addresses the variable most strongly and centrally associated with override intention in these data. The decision-curve analysis reported in Table 9 and the thresholds it provides are exploratory and illustrate, in principle, how such factors might inform unit-level prioritization rather than individual-level screening, in line with the original framework for clinical utility evaluation of prediction tools [24]. Addressing moral distress through structured ethics-of-AI debriefs and protected reflection time may be relevant to the intermediate association between digital workload and override intention, particularly in light of the unique ethical and legal consequences that artificial intelligence introduces into nursing practice [25]. Strengthening psychological safety at the unit level—through leadership behaviors, structured speak-up routines, and shared accountability frameworks—may be associated with a weaker overall pattern of strain, an approach grounded in longstanding nursing ethics scholarship in which constrained moral agency is recognized as a major contributor to distress and attrition [26]. The exploratory multivariable model achieved acceptable internal discrimination (c = 0.78) and positive net benefit across pragmatic thresholds; however, it was internally validated in a single center and predicts a self-reported intention rather than observed behavior, and should therefore be regarded as preliminary. Designating individual nurses as high-risk on the basis of a self-reported intention scale raises ethical concerns relating to surveillance and individual blame. Any translational use would require prospective, multicenter validation against objective override data.

The magnitude of the observed associations warrants emphasis alongside their statistical significance. The standardized coefficients for alert fatigue (β = 0.32) and moral distress (β = 0.24) represent small-to-moderate associations, and the full multivariable model explained approximately one-third of the variance in override intention (R^2^ = 0.34), leaving most variance unexplained. A 5-point increase in alert fatigue corresponded to an estimated 1.6-point increase on the 8–40 override intention scale, and the index of moderated mediation, although statistically significant, was numerically small (−0.014). These effect sizes indicate that the modifiable factors examined here are meaningful but partial correlates that should be situated alongside other clinical, technological, and organizational influences not measured in this study.

Generalizability is constrained by the single-center setting and the early, partial phase of AI-DSS implementation, in which fewer than one in five nurses had received structured exposure to the tools. Settings with mature, fully embedded AI-DSS, different alert architectures, different staffing ratios, or different scope-of-practice regulations may show different associations; where AI alerts are pervasive and high-stake, alert fatigue and its correlates could be more pronounced, whereas well-curated, high-specificity alerts could attenuate them. The relative novelty of these tools in the present cohort may also mean that responses reflect general attitudes toward AI in addition to experience-based reactions. Multicenter studies spanning a range of AI-integration maturity levels are therefore needed before the prevalence of the profiles or the strength of the associations can be extrapolated to other systems.

### 4.2. Study Limitations

Several limitations should frame interpretation. First, the cross-sectional design precludes temporal or causal inference; the moderated mediation results should be interpreted as a statistical decomposition of associations rather than as evidence of directional effects. Second, all measures were collected through self-report in a single survey session, raising potential common-method bias despite procedural safeguards such as anonymous administration, separation of conceptually distinct constructs across instruments, and embedded attention checks; this concern is particularly relevant for behaviorally proximal outcomes such as missed-care indicators and process-level care reliability, which have been shown to correlate with workload and team variables in ways that may partly overlap with our constructs [27]. Third, the study was conducted in a single Romanian tertiary hospital, which may limit transferability to settings with mature AI deployment, different staffing models, or different scope-of-practice regulations; broader multi-country evidence on neglected care suggests substantial cross-system variability in the work-system determinants of nursing process outcomes [28]. Fourth, AI-DSS override intention was measured as a behavioral disposition rather than as documented override behavior; objective override logs were not available, and the construct should not be conflated with observed clinical action—a distinction increasingly emphasized in recent health-system surveys of AI adoption, in which perceived priorities, real-world adoption, and downstream impact are recognized as separate constructs [29]. Exposure to AI-DSS was heterogeneous and only partially documented: the tools were implemented incrementally and unevenly, only 19.7% of nurses had structured exposure, and logs of alert type, frequency, and clinical context were unavailable, so the outcome may reflect a combination of experience-based responses and more general attitudes toward AI. The upper-tertile definition of high override intention is based on a self-report scale rather than behavior or a clinical outcome, and carries the ethical sensitivities noted above. Because data were obtained from a single self-administered session, common-method variance and socially desirable responses may have affected the estimates; the right-skewed distribution of override intention is compatible with some under-reporting. Four instruments were adapted or newly developed and, although translation, content validity, and factor-analytic evidence are reported, they are not yet externally validated, so the reported psychometrics should be considered preliminary. Fifth, the latent profile analysis and network analysis were partly exploratory; although fit indices supported the three-profile solution and bootstrap CS-coefficients indicated acceptable edge stability, external validation in independent cohorts is needed before clinical adoption. Sixth, our sleep disturbance instrument was a brief proxy rather than a full validated measure, and substantive variance in sleep architecture may remain unmeasured relative to comprehensive instruments such as the Pittsburgh Sleep Quality Index [30]. Future longitudinal multicenter studies that combine validated self-reports with objective override and workflow data will be essential for confirming these hypothesis-generating associations.

## 5. Conclusions

In this cross-sectional cohort, self-reported AI-DSS override intention varied substantially and clustered into three interpretable work-system profiles aligned with trust-calibration tendencies: algorithm appreciators, calibrated trusters, and algorithm-averse nurses. The algorithm-averse profile, comprising approximately one-third of the cohort, was characterized by simultaneously elevated alert fatigue, moral distress, cognitive load, and sleep disturbance, and by reduced psychological safety and resilience. In multivariable analysis, alert fatigue, moral distress, psychological safety, cognitive load, and resilience were independent correlates of override intention, while demographic factors were not. Moral distress statistically accounted for a substantial portion of the cross-sectional alert fatigue–override intention association, and the strength of this indirect association varied with psychological safety, with the partial correlation network analysis localizing alert fatigue as the most central node of the multivariate structure. An exploratory multivariable model achieved acceptable internal discrimination and positive net benefit across pragmatic threshold probabilities. These findings are associative and hypothesis-generating, concern intention rather than observed behavior, and require prospective, multicenter validation against objective override data before any screening or intervention-targeting application is considered.

## Figures and Tables

**Table 1:** Participant characteristics by shift configuration (day-only vs. rotating/night).

Characteristic	Overall (*n* = 239)	Day-Only (*n* = 147)	Rotating/Night (*n* = 92)	*p*-Value	Test
Age (years), mean ± SD	33.7 ± 8.2	34.6 ± 8.1	32.3 ± 8.3	0.037	*t*-test
Female, *n* (%)	183 (76.6)	118 (80.3)	65 (70.7)	0.082	χ^2^
Bachelor’s or higher, *n* (%)	161 (67.4)	104 (70.7)	57 (61.9)	0.156	χ^2^
Experience (years), median (IQR)	8 (4–13)	9 (5–14)	6 (3–11)	0.018	Mann–Whitney
Critical care unit, *n* (%)	60 (25.1)	29 (19.7)	31 (33.7)	0.013	χ^2^
Prior structured AI-DSS exposure, *n* (%)	47 (19.7)	34 (23.1)	13 (14.1)	0.083	χ^2^
Alert fatigue score, mean ± SD	28.4 ± 8.7	26.7 ± 8.3	31.1 ± 8.6	<0.001	*t*-test
Moral distress score, mean ± SD	22.6 ± 7.4	21.3 ± 7.1	24.7 ± 7.4	0.001	*t*-test
Psychological safety, mean ± SD	23.1 ± 4.6	23.8 ± 4.4	22.0 ± 4.7	0.003	*t*-test
Sleep disturbance, mean ± SD	9.7 ± 3.4	8.9 ± 3.2	10.9 ± 3.4	<0.001	*t*-test
AI-DSS override intention, mean ± SD	14.3 ± 6.8	13.1 ± 6.4	16.2 ± 6.9	<0.001	*t*-test

**Table 2:** Scale scores, theoretical ranges, and internal consistency (Cronbach’s α, McDonald’s ω, and composite reliability [CR]).

Instrument (Items)	Possible Range	Mean ± SD	Median (IQR)	Skewness	Cronbach α	McDonald’s ω (CR)
Alert-Fatigue Scale (12)	12–60	28.4 ± 8.7	27 (22–34)	0.31	0.87	0.88 (0.87)
Moral Distress Scale–R brief (10)	10–50	22.6 ± 7.4	22 (17–28)	0.43	0.83	0.85 (0.84)
Cognitive Load brief NASA-TLX (6)	6–60	34.7 ± 9.8	35 (28–42)	−0.08	0.79	0.80 (0.79)
Sleep Disturbance (5)	0–21	9.7 ± 3.4	10 (7–12)	0.22	0.74	0.76 (0.75)
Edmondson Psychological Safety (7)	7–35	23.1 ± 4.6	23 (20–26)	−0.18	0.81	0.83 (0.82)
CD-RISC-10 Resilience (10)	0–40	26.8 ± 5.9	27 (23–31)	−0.27	0.86	0.87 (0.86)
AI-DSS Override Intention (8)	8–40	14.3 ± 6.8	13 (9–19)	0.62	0.84	0.86 (0.85)
Trust-Calibration Tendency (5)	5–25	15.6 ± 3.8	16 (13–18)	−0.11	0.78	0.80 (0.79)

Note. α = Cronbach’s alpha; ω = McDonald’s omega; CR = composite reliability, computed from standardized confirmatory factor loadings for the four adapted or newly developed instruments (Alert-Fatigue Scale, Moral Distress Scale–R brief, AI-DSS Override Intention, and Trust-Calibration Tendency). McDonald’s ω is reported for all multi-item scales; CR is reported only for the adapted or newly developed scales, for which a confirmatory factor model was estimated. All coefficients met the conventional ≥0.70 criterion.

**Table 3:** Key outcomes across tertiles of alert fatigue.

Variable	Low AF (*n* = 82)	Medium AF (*n* = 79)	High AF (*n* = 78)	*p*-Trend	Test
Alert fatigue score	19.7 ± 3.1	27.6 ± 2.4	38.1 ± 4.6	<0.001	ANOVA
Moral distress	18.4 ± 6.1	22.3 ± 6.7	27.2 ± 7.2	<0.001	ANOVA
Cognitive load	29.6 ± 8.4	34.1 ± 9.1	40.3 ± 9.2	<0.001	ANOVA
Psychological safety	24.6 ± 4.3	23.0 ± 4.5	21.7 ± 4.6	<0.001	ANOVA
Resilience (CD-RISC-10)	28.3 ± 5.6	26.7 ± 5.7	25.4 ± 6.1	0.002	ANOVA
Sleep disturbance	8.1 ± 2.8	9.8 ± 3.3	11.3 ± 3.4	<0.001	ANOVA
Override intention	9.4 ± 4.1	13.7 ± 5.2	18.9 ± 6.3	<0.001	ANOVA
Trust-calibration tendency	16.8 ± 3.4	15.4 ± 3.7	14.6 ± 3.9	<0.001	ANOVA
Intention to leave (12 m), *n* (%)	11 (13.4)	21 (26.6)	33 (42.3)	<0.001	χ^2^ trend

**Table 4:** Pearson correlations among study variables (*n* = 239). All *p*-values from two-sided tests.

Variable	1	2	3	4	5	6	7	8
1. Alert fatigue	—	0.43 **	0.46 **	−0.27 **	−0.21 **	0.34 **	0.38 **	−0.29 **
2. Moral distress		—	0.37 **	−0.31 **	−0.18 *	0.26 **	0.31 **	−0.24 **
3. Cognitive load			—	−0.22 **	−0.17 *	0.28 **	0.29 **	−0.19 *
4. Psychological safety				—	0.29 **	−0.23 **	−0.18 *	0.34 **
5. Resilience					—	−0.31 **	−0.22 **	0.27 **
6. Sleep disturbance						—	0.21 *	−0.16 *
7. Override intention							—	−0.41 **
8. Trust-calibration tendency								—

Note: * *p* < 0.05; ** *p* < 0.01.

**Table 5:** Multivariable linear regression predicting AI-DSS override intention (*n* = 239).

Predictor	β (unstd.)	SE	Std. β	95% CI	*p*-Value	VIF
Alert fatigue (per 5 pts)	1.62	0.34	0.32	0.96 to 2.29	<0.001	1.62
Moral distress (per 5 pts)	1.14	0.36	0.24	0.43 to 1.86	0.002	1.41
Cognitive load (per 5 pts)	0.47	0.21	0.13	0.06 to 0.89	0.026	1.49
Psychological safety (per 5 pts)	−1.06	0.41	−0.17	−1.86 to −0.26	0.009	1.27
Resilience (per 5 pts)	−0.71	0.33	−0.13	−1.36 to −0.06	0.033	1.18
Sleep disturbance (per 3 pts)	0.39	0.18	0.11	0.04 to 0.74	0.029	1.31
Critical care unit (vs. ward)	1.83	0.86	0.13	0.13 to 3.53	0.036	1.22
Rotating/night shift (vs. day)	1.27	0.79	0.10	−0.29 to 2.84	0.108	1.34
Female sex	−0.43	0.91	−0.03	−2.22 to 1.36	0.638	1.07
Age (per 5 years)	−0.16	0.27	−0.04	−0.69 to 0.37	0.557	1.18
Bachelor’s or higher education	−0.62	0.81	−0.04	−2.22 to 0.98	0.443	1.09

Model R^2^ = 0.34; adjusted R^2^ = 0.31; F(11, 227) = 10.7, *p* < 0.001. All VIF < 2.0.

**Table 6:** Multinomial logistic regression for trust-calibration phenotype membership (reference = calibrated trusters, *n* = 98).

Predictor (Per 5-Point Increase)	Appreciators vs. Calibrated OR (95% CI)	*p*-Value	Averse vs. Calibrated OR (95% CI)	*p*-Value
Alert fatigue	0.67 (0.51–0.88)	0.004	1.71 (1.31–2.23)	<0.001
Moral distress	0.82 (0.63–1.07)	0.146	1.46 (1.13–1.89)	0.004
Cognitive load	0.91 (0.74–1.12)	0.379	1.27 (1.04–1.55)	0.019
Psychological safety	1.18 (0.91–1.53)	0.218	0.73 (0.57–0.93)	0.011
Resilience	1.21 (0.95–1.54)	0.119	0.81 (0.64–1.02)	0.073
Sleep disturbance	0.89 (0.71–1.12)	0.317	1.24 (1.01–1.53)	0.043
Critical care unit (yes vs. no)	0.84 (0.43–1.64)	0.611	1.62 (0.91–2.89)	0.103
Rotating/night shift	0.71 (0.39–1.30)	0.268	1.43 (0.84–2.43)	0.187

Model log-likelihood = −207.4; pseudo-R^2^ (Nagelkerke) = 0.31; classification accuracy = 61.9%.

**Table 7:** Latent profile analysis: fit indices and class assignment for 2–5-profile solutions.

Profiles (k)	LL	BIC	SABIC	Entropy	BLRT *p*	Smallest Class %	Decision
2	−2147.6	4382.4	4319.7	0.71	<0.001	37.2	Rejected
3	−2078.3	4279.8	4192.6	0.78	<0.001	26.8	Selected
4	−2061.4	4291.2	4179.5	0.74	0.213	12.1	Rejected
5	−2049.1	4310.9	4174.7	0.69	0.487	7.5	Rejected

Note. LL = log-likelihood; BIC = Bayesian Information Criterion; SABIC = sample-size adjusted BIC; BLRT = bootstrapped likelihood ratio test (*n* = 1000 bootstrap samples). Lower BIC/SABIC and entropy > 0.70 favor the selected solution.

**Table 8:** Moderated mediation: alert fatigue → moral distress → AI-DSS override intention, moderated by psychological safety (bootstrap *n* = 5000).

Path	Estimate	Boot SE	95% Bias-Corrected CI	*p*-Value
a (alert fatigue → moral distress)	0.349	0.058	0.236 to 0.464	<0.001
b (moral distress → override) at mean PS	0.218	0.072	0.077 to 0.358	0.003
c′ (direct effect alert fatigue → override)	0.213	0.046	0.123 to 0.301	<0.001
Indirect via moral distress (overall)	0.108	0.026	0.063 to 0.166	<0.001
Indirect at −1 SD psychological safety	0.142	0.034	0.083 to 0.214	<0.001
Indirect at mean psychological safety	0.081	0.022	0.043 to 0.128	<0.001
Indirect at +1 SD psychological safety	0.041	0.018	0.011 to 0.083	0.018
Index of moderated mediation	−0.014	0.005	−0.024 to −0.005	0.004
Total effect (alert fatigue → override)	0.321	0.044	0.235 to 0.408	<0.001
Proportion mediated (overall)	33.6%	—	—	—

All paths are standardized regression coefficients adjusted for age, sex, unit type, and shift configuration. Index of moderated mediation reflects change in indirect effect per 1-SD increase in psychological safety.

**Table 9:** Exploratory operating characteristics and decision-curve metrics for identifying nurses at elevated risk of high override intention (top tertile, *n* = 78).

Threshold Prob.	Sensitivity	Specificity	PPV	NPV	F1 Score	Net Benefit	Δ vs. Treat-All
0.08	0.946	0.234	0.380	0.918	0.541	0.187	+0.043
0.12	0.918	0.391	0.434	0.911	0.591	0.176	+0.061
0.17 (optimal)	0.831	0.547	0.487	0.871	0.616	0.143	+0.072
0.22	0.762	0.683	0.547	0.852	0.638	0.118	+0.061
0.28	0.687	0.769	0.602	0.823	0.642	0.087	+0.029
0.34	0.561	0.846	0.661	0.794	0.607	0.063	+0.012
0.40	0.487	0.892	0.703	0.776	0.576	0.041	+0.004
Brier score = 0.142							
Calibration slope = 0.93							
Hosmer–Lemeshow *p* = 0.41							
C-statistic (AUC) = 0.781							

Operating characteristics and net benefit were calculated using 10-fold internal cross-validation. PPV = positive predictive value; NPV = negative predictive value.

## Data Availability

The data presented in this study are available upon request from the corresponding author.
